# Clonal Evolution of *Enterocytozoon bieneusi* Populations in Swine and Genetic Differentiation in Subpopulations between Isolates from Swine and Humans

**DOI:** 10.1371/journal.pntd.0004966

**Published:** 2016-08-26

**Authors:** Qiang Wan, Lihua Xiao, Xichen Zhang, Yijing Li, Yixin Lu, Mingxin Song, Wei Li

**Affiliations:** 1 College of Veterinary Medicine, Northeast Agricultural University, Harbin, Heilongjiang, China; 2 Division of Foodborne, Waterborne and Environmental Diseases, National Center for Emerging and Zoonotic Infectious Diseases, Centers for Disease Control and Prevention, Atlanta, Georgia, United States of America; 3 College of Veterinary Medicine, Jilin University, Changchun, Jilin, China; Temple University, UNITED STATES

## Abstract

*Enterocytozoon bieneusi* is a widespread parasite with high genetic diversity among hosts. Its natural reservoir remains elusive and data on population structure are available only in isolates from primates. Here we describe a population genetic study of 101 *E*. *bieneusi* isolates from pigs using sequence analysis of the ribosomal internal transcribed spacer (ITS) and four mini- and microsatellite markers. The presence of strong linkage disequilibrium (LD) and limited genetic recombination indicated a clonal structure for the population. Bayesian inference of phylogeny, structural analysis, and principal coordinates analysis separated the overall population into three subpopulations (SP3 to SP5) with genetic segregation of the isolates at some geographic level. Comparative analysis showed the differentiation of SP3 to SP5 from the two known *E*. *bieneusi* subpopulations (SP1 and SP2) from primates. The placement of a human *E*. *bieneusi* isolate in pig subpopulation SP4 supported the zoonotic potential of some *E*. *bieneusi* isolates. Network analysis showed directed evolution of SP5 to SP3/SP4 and SP1 to SP2. The high LD and low number of inferred recombination events are consistent with the possibility of host adaptation in SP2, SP3, and SP4. In contrast, the reduced LD and high genetic diversity in SP1 and SP5 might be results of broad host range and adaptation to new host environment. The data provide evidence of the potential occurrence of host adaptation in some of *E*. *bieneusi* isolates that belong to the zoonotic ITS Group 1.

## Author Summary

This study explored the genetic characteristics of the ITS and four mini- and microsatellite markers and assessed the population structure and substructures in 101 *E*. *bieneusi* isolates from pigs in China. The measures of LD and recombination events supported the occurrence of clonal evolution among the isolates from four study areas of China. Three subpopulations (SP3 to SP5) with potentially varied host ranges were identified within the isolates, which were genetically differentiated from two existing primate subpopulations SP1 and SP2. Population genetic analysis indicated that the isolates in SP1 and SP5 with clonal structure might be responsible for the cross-species transmission and thus have zoonotic potential, while the isolates in SP2, SP3, and SP4 with epidemic structure or host-adapted traits might colonize specific hosts. The data revealed the presence of clonality, potential host adaptation, and population differentiation of *E*. *bieneusi* in different hosts.

## Introduction

Microsporidia are obligate intracellular eukaryotic parasites that infect a wide range of animals and are closely related to fungi [1,2]. Genome analyses conducted in several recent studies strongly suggest that some species of microsporidia could have a diploid or polyploid stage and a sexual cycle, and might be true Fungi [3–8]. Nevertheless, the ploidy level of *Enterocytozoon bieneusi* and whether it undergoes mating and a meiotic cycle are still unclear. *E*. *bieneusi* is the most common human microsporidian species and can colonize a variety of other mammals and birds [2,9]. This ubiquitous pathogen causes diarrhea of various severity and duration in relation to host immune status [2,10]. Genotyping of isolates has improved our understanding of the genetic characteristics and the potential transmission modes of *E*. *bieneusi* among hosts. *E*. *bieneusi* exhibits high genetic diversity among isolates from different hosts [11,12]. Over 200 *E*. *bieneusi* genotypes have been identified in humans, companion animals, livestock, horses, birds, and wildlife based on DNA sequence analysis of the ribosomal internal transcribed spacer (ITS) and the established naming convention [13,14]. The genotypes form several genetically isolated clusters (Groups 1 to 8) in phylogenetic analysis, with some found in specific host groups [11,12,15]. Humans and pigs are mainly infected with the zoonotic Group 1 genotypes, ruminants with host-adapted Group 2 genotypes, and dogs with the genotypes in an outlier group [2,16–22].

The public health potential of *E*. *bieneusi* in animals has been assessed in numerous studies and pigs were recognized as the most significant reservoir [2,17,23]. However, this was based on results of sequence analysis of a single ITS marker (392 bp in length), which may not adequately represent the evolutionary history of the *E*. *bieneusi* genome with a length of about 6 Mb [24]. Several mini- and microsatellites with sufficient resolution have been available to infer subgroup-level phylogenies [25]. Coupled with the ITS locus, they have been used effectively in characterizations of population structures and substructures of *E*. *bieneusi* in primates [26–28]. In these studies, a clonal structure was found in human *E*. *bieneusi* populations from Peru, India, and Nigeria and no apparent geographic segregation of the isolates was observed. Nevertheless, two genetically isolated subpopulations (SP1 and SP2) were identified within the overall population. Significant linkage disequilibrium (LD) and limited recombination in SP1 support a clonal population structure, while the rapid expansion of some specified multilocus genotypes (MLGs) in SP2 obscures the limited genetic exchange because of its epidemic population structure [26,27]. Although the isolates used in SP1 and SP2 mainly belong to ITS Group 1 genotypes D, IV, and A with zoonotic potential, some of them displayed host-specific features when mini- and microsatellites were considered in the analysis [26,27]. Population genetic traits of *E*. *bieneusi* in non-human primates from China were similar to those in humans [28]. These observations on the population genetics of *E*. *bieneusi* in primates need to be substantiated in other hosts.

The objectives of this study were to characterize 101 pig *E*. *bieneusi* isolates that belong to nine genotypes in zoonotic Group 1 at the subtype level at five genetic loci, to assess the population structure and substructures, and to compare them with similar data from *E*. *bieneusi* subpopulations SP1 and SP2 in primates to examine the occurrence and extent of host segregation in different *E*. *bieneusi* subpopulations.

## Materials and Methods

### Ethics statement

This study was performed in accordance with the recommendations in the Guide for the Care and Use of Laboratory Animals of the Ministry of Health, China. Prior to experiments, the protocol of the current study was reviewed and approved by the Institutional Animal Care and Use Committee of Northeast Agricultural University (approved protocol number SRM-08). For specimen collection, we obtained permission from animal owners. No specific permits were required for the described field studies, and the locations where we sampled are not privately owned or protected in any way. The field studies did not involve endangered or protected species.

### Study area and parasite sampling

Isolates of *E*. *bieneusi* were obtained from pigs in cities Changchun, Daqing, Harbin, and Qiqihar in northeast China. All of them were previously genotyped by PCR and sequence analysis of the ITS locus [17,29]. A total of 101 isolates of ITS genotypes CHN7, CS-4, EbpA, EbpB, EbpC, Henan-I, Henan-IV, O, and PigEBITS3 in zoonotic Group 1 were selected for population genetic analysis in this study. The number of isolates and their ITS genotype designations by city are shown in Table 1. For comparative purposes, population genetic data from 101 *E*. *bieneusi* isolates in humans from India, Peru, and Nigeria and 5 isolates in captive baboons from Kenya were included in this analysis (Table 1) [26,27].

**Table 1:** *Enterocytozoon bieneusi* isolates analyzed in this study and their genotypes based on the ribosomal internal transcribed spacer (ITS) sequences.

Location	Host	No.	ITS genotype (no., city)	Reference
China	Pigs	101	CHN7 (3, Changchun), CS-4 (27, Harbin), EbpA (18, Changchun; 4, Qiqihar), EbpB (12, Daqing), EbpC (11, Changchun; 10, Daqing; 2, Harbin; 9, Qiqihar), Henan-I (1, Changchun), Henan-IV (2, Qiqihar), O (1, Qiqihar), PigEBITS3 (1, Changchun)	[17,29]
India	HIV^+^ children	14	A (3), D (5), PigEBITS7 (6)	[26]
Nigeria	HIV^+^ adults	15	A (6), D (2), IV (6), Nig2 (1)	[26]
Peru	HIV^+^ adults	72	A (30), D (8), EbpC (1), IV (16), Peru7 (5), Peru8 (1), Peru10 (3), Peru11 (4), WL11 (4)	[27]
Kenya	Olive baboons	5	D (5)	[26]

### PCR and sequencing

Genomic DNAs extracted from 101 pig fecal specimens were used for PCR amplification of a minisatellite marker MS4 and three microsatellite markers (MS1, MS3, and MS7) as described [25]. The secondary PCR products with the expected sizes (bp) of approximately 676 for MS1, 537 for MS3, 885 for MS4, and 471 for MS7 were sequenced in both directions at the Beijing Genomics Institute, China. Raw sequences were assembled and edited with the software Chromas Pro version 1.33 (Technelysium Pty. Ltd., Helensvale, Queensland, Australia). The sequences obtained were compared to the sequence data of each target available in GenBank [26,27] using the software MAFFT version 7.300 (http://mafft.cbrc.jp/alignment/software/) [30].

### Genetic analysis

The software DnaSP version 5.10.01 (http://www.ub.edu/dnasp/) was used to determine *E*. *bieneusi* genotypes at each of the five markers and the MLGs with consideration of both single base nucleotide substitutions and short insertions and deletions (indels) polymorphisms [31]. Intragenic LD and recombination rates for individual locus and concatenated multilocus data set were estimated from the segregating sites without consideration of indels using DnaSP [31]. Recombination rates were further assessed using the methods GENECONV, MaxChi, and SiScan implemented in the software RDP version 3.44 (http://darwin.uvigo.es/rdp/rdp.html) [32]. Tests for genetic diversity and neutrality (Fu’s Fs and Tajima’s D) were run on the concatenated contigs using DnaSP (based on segregating sites) and the software Arlequin 3.5.1.2 (http://cmpg.unibe.ch/software/arlequin35/; based on both segregating sites and indels) [31,33]. The different nucleotide sequences (considering both substitutions and indels) were assigned as distinct alleles and the alleles at each of the five loci defined the allelic profile or sequence type. We measured the pairwise intergenic LD on annotated allelic profiles using the exact test and Markov chain parameters implemented in Arlequin [33]. Values of standardized index of association (*I*^S^_A_) were calculated with LIAN 3.5 (http://guanine.evolbio.mpg.de/cgi-bin/lian/lian.cgi.pl/query) on five-loci haplotypes [34].

We assessed population structure of *E*. *bieneusi* by analyzing the intragenic and intergenic LD, *I*^S^_A_, neutrality, and recombination events (Rms). Wright’s fixation index (*F*_ST_) calculated using Arlequin and gene flow (*Nm*) calculated using DnaSP were applied to evaluate the degree of genetic differentiation between *E*. *bieneusi* populations [31,33].

### Phylogenetic and structure sub-clustering analysis

A Bayesian analysis implemented in the software MrBayes version 3.2.1 (http://mrbayes.sourceforge.net/) was used in clustering nucleotide sequences using Markov chain Monte Carlo (MCMC) methods [35]. The general time reversible model (GTR+G) was determined to be the best-fit nucleotide substitution model with the program ModelTest 3.7 (http://www.molecularevolution.org/software/phylogenetics/modeltest) [36]. An MCMC-based analysis of phylogeny was conducted using the GTR+G model and the default parameters settings as described [26]. The maximum clade credibility tree generated by these analyses was visualized and edited using the software FigTree version 1.3.1 (http://tree.bio.ed.ac.uk/software/figtree/). Pairwise distance matrices among nucleotide sequences of MLGs were calculated using eachgap calculating method with the dist.seqs command in the software MOTHUR version 1.24.1 (http://www.mothur.org/wiki/Download_mothur) [37]. A principal coordinates analysis (PCoA) via covariance matrix with data standardization was performed on the generated matrices with the software GENALEX version 6.501 (http://biology-assets.anu.edu.au/GenAlEx) [38]. A Bayesian cluster analysis was performed on the allelic profile data using the software STRUCTURE version 2.3.1 (http://pritch.bsd.uchicago.edu/software.html) to assess the presence of distinct subpopulations [39]. We also constructed haplotype networks using the median-joining method implemented in the software Network version 4.6.1.1 (http://www.fluxus-engineering.com/sharenet_rn.htm) to estimate the genetic segregation and evolutionary trend of *E*. *bieneusi* isolates [40].

## Results

### Genotyping and LD at individual loci

Sequence analysis of 101 pig *E*. *bieneusi* isolates identified 9, 15, 5, 15, and 11 genotypes at the loci ITS, MS1, MS3, MS4, and MS7, respectively. Single nucleotide polymorphisms (SNPs) were the only source of genetic diversity at the ITS locus, while genetic variation at the other four loci included the lengths of trinucleotide TAC and TAA repeats at MS1, dinucleotide TA repeats at MS3, tetranucleotide GGTA repeats at MS4, and TAA repeats at MS7 and the SNPs outside the tandem repeat regions. Some MS4 fragments also carried isostructural GG to AA substitutions in the first tetranucleotide repeat. Gene diversity (Hd) at individual loci was calculated using DnaSP and is shown in Table 2. The number of genotypes and Hd value of each locus were also measured for 106 primate *E*. *bieneusi* isolates (Table 2). Generally, markers MS1 and MS4 had higher resolution than the other ones (Table 2). The intragenic LD among segregating sites for each locus was calculated based on a linear regression analysis in DnaSP. The markers MS1, MS3, and MS7 had complete LD (LD = 1) when only the pig *E*. *bieneusi* isolates were analyzed, whereas only MS1 and MS3 had complete LD in the analysis of all 207 isolates (Table 3). Table 3 displays the number of pairwise comparisons and the number of significant pairwise comparisons after Fisher’s exact test and Bonferroni correction. The occurrence of intragenic recombination was assessed using DnaSP. As shown in Table 3, genetic recombination was only detected in markers with incomplete LD.

**Table 2:** Number of *Enterocytozoon bieneusi* genotypes based on combined sequence length and nucleotide polymorphism at four mini- and microsatellite loci.

City/subpopulation	No. of isolate	No. of genotypes (gene diversity)				
		MS1	MS3	MS4	MS7	ITS
Changchun	34	6 (0.69)	4 (0.61)	7 (0.76)	6 (0.77)	5 (0.62)
Daqing	22	3 (0.62)	2 (0.52)	5 (0.65)	3 (0.56)	2 (0.52)
Harbin	29	4 (0.53)	3 (0.31)	3 (0.26)	3 (0.14)	2 (0.13)
Qiqihar	16	4 (0.59)	1 (0)	3 (0.70)	4 (0.35)	4 (0.64)
Total (4 cities)	101	15 (0.90)	7 (0.66)	15 (0.85)	11 (0.81)	9 (0.77)
SP1	66	35 (0.96)	10 (0.78)	9 (0.79)	15 (0.87)	9 (0.79)
SP2	39	4 (0.28)	2 (0.15)	5 (0.33)	3 (0.23)	1 (0)
SP3	35	9 (0.77)	5 (0.62)	9 (0.76)	7 (0.83)	3 (0.50)
SP4	41	9 (0.74)	4 (0.27)	5 (0.56)	6 (0.56)	5 (0.52)
SP5	26	5 (0.66)	4 (0.40)	7 (0.80)	5 (0.57)	2 (0.21)
Total (5 subpopulations)	207	54 (0.94)	17 (0.84)	29 (0.92)	24 (0.91)	19 (0.89)

**Table 3:** Intragenic linkage disequilibrium and recombination events at individual genetic loci.

Locus	*S*	*P*	*F*	*B*	LD (|D'|)	Rm
MS1^a^	3	3	1	1	1	0
MS3^a^	3	3	0	0	1	0
MS4^a^	21	210	170	137	0.9716–0.1250X	5
MS7^a^	3	3	1	1	1	0
ITS^a^	12	66	36	32	0.9924–0.0137X	2
MS1^b^	7	21	4	4	1	0
MS3^b^	8	28	9	5	1	0
MS4^b^	35	595	331	279	0.9951–0.0317X	4
MS7^b^	20	190	88	79	0.9724 + 0.1318X	2
ITS^b^	20	190	81	52	0.9641 + 0.0053X	3

*S*: number of segregating sites; *P*: number of pairwise comparisons; *F*: number of significant pairwise comparisons by Fisher’s exact test; *B*: number of significant comparisons after the Bonferroni correction; LD (|D’|): linkage disequilibrium between sites and X is the nucleotide distance (measured in kilobases; kb); Rm: minimum number of recombination events.

^a^Among 101 *E*. *bieneusi* isolates from pigs.

^b^Among 207 *E*. *bieneusi* isolates from pigs, humans, and baboons.

### Multilocus sequence typing (MLST) and analysis

To investigate the genetic diversity and population characteristics of *E*. *bieneusi*, the five loci were concatenated into a single multilocus contig of 2,128 bp in length. The contigs from 101 pig isolates include 159 polymorphic sites (41 segregating sites and 118 indel sites). Due to the difference in substitution rate between SNPs and indels, two methods were used to estimate genetic diversity. The finite population genetic variance estimates that consider both SNPs and indels allowed identification of a total of 44 MLGs with a Hd value of 0.95 and a nucleotide diversity (Pi) value of 0.0197 (Table 4). In contrast, the use of infinite population genetic variance estimates that consider only SNPs led to reduced genetic diversity (MLGs = 37, Hd = 0.93, Pi = 0.0065) (Table 4). The genetic diversity was also estimated for each of the four cities in northeast China (Table 4).

**Table 4:** Genetic diversity in *Enterocytozoon bieneusi* based on the analysis of concatenated sequences from five genetic loci.

Taxa	Test model	Variability of multilocus gene sequences										
		*N*	Hd	*k*	Pi	Theta (*k*)	Fs (Obs)	*P* (Fs ≤ Obs)	D (Obs)	*P* (D ≤ Obs)	LD (|D'|)	Rm
Changchun	*F*	19	0.93	25	0.0117	25	2.14	0.830	2.19	0.994		
	*I*	16	0.91	13	0.0063	13	0.84	0.655	2.21	0.993	1.0024–0.1373X	4
Daqing	*F*	8	0.82	56	0.0263	56	19.23	1.000	1.16	0.908		
	*I*	7	0.75	11	0.0056	11	6.24	0.980	1.16	0.913	0.9635–0.1451X	4
Harbin	*F*	8	0.61	9	0.0041	9	4.86	0.961	**−1.12**	0.121		
	*I*	6	0.37	3	0.0015	3	1.84	0.813	**−1.10**	0.146	0.9988–0.1797X	3
Qiqihar	*F*	9	0.86	29	0.0138	29	5.66	0.993	1.61	0.977		
	*I*	8	0.85	10	0.0050	10	2.37	0.843	1.52	0.961	1.0282–0.2990X	2
Total (4 cities)	*F*	44	0.95	42	0.0197	42	3.26	0.835	2.10	0.978		
	*I*	37	0.93	13	0.0065	13	**−3.38**	0.248	2.07	0.983	0.9352–0.2020X	11
SP1	*F*	51	0.99	31	0.0142	31	−10.35	0.025	0.04	0.583		
	*I*	33	0.97	6	0.0029	6	−15.73	0.000	0.05	0.580	0.9473–0.1084X	5
SP2	*F*	9	0.52	80	0.0388	80	∞	N/A	−0.97	0.188		
	*I*	7	0.51	8	0.0051	8	7.68	0.983	−0.92	0.175	1.0165–0.1078X	2
SP3	*F*	18	0.91	53	0.0250	53	9.33	0.994	1.55	0.957		
	*I*	17	0.91	11	0.0055	11	−0.21	0.510	1.66	0.963	0.9724–0.0385X	5
SP4	*F*	14	0.79	16	0.0071	16	4.80	0.972	−0.96	0.168		
	*I*	11	0.67	4	0.0018	4	−0.68	0.427	−0.87	0.206	0.9844–0.0339X	3
SP5	*F*	13	0.90	15	0.0069	15	2.18	0.840	0.01	0.556		
	*I*	10	0.84	4	0.0020	4	−0.60	0.416	0.01	0.546	0.9399 + 0.0413X	3
Total (5 subpopulations)	*F*	105	0.97	72	0.0328	72	**−1.64**	0.483	**−0.55**	0.349		
	*I*	76	0.96	13	0.0080	13	**−27.05**	0.001	**−0.54**	0.339	0.9773–0.0401X	13

*F*: finite population genetic variance estimates; *I*: infinite population genetic variance estimates; *N*: number of multilocus genotypes; Hd: gene diversity; *k*: mean number of pairwise differences; Pi: nucleotide diversity (average over loci); Theta (*k*): gene variance based on mean number of pairwise differences; Fs/D (Obs): observed value of Fu’s/Tajima’s statistic testing selective neutrality based on allele frequency; *P* (Fs/D ≤ Obs): probability of obtaining Fs/D value equal or lower than the observed; LD (|D'|): linkage disequilibrium between sites and X is the nucleotide distance (measured in kilobases; kb); Rm: minimum number of recombination events.

Tests for intragenic LD and recombination among segregating sites were performed on combined multilocus contigs. The overall population and individual populations of *E*. *bieneusi* in pigs from four geographic locations all had significant but incomplete LD (Table 4). The negative slope returned by LD score regression is indicative of LD index declines with increasing nucleotide distance, implying the potential occurrence of recombination. A varying number of Rms were detected in the overall and individual pig *E*. *bieneusi* populations from four cities using DnaSP (Table 4). The occurrence of Rms was also confirmed using the GENECOV, MaxChi, and SiScan methods in RDP4 (S1 Table). The neutrality tests conducted with DnaSP (using SNPs only) and Arlequin (using both SNPs and indels) were both significant, rejecting the null hypothesis of a neutral population at mutation-drift equilibrium (Table 4). The negative Fs and D values (highlighted in bold in the center of Table 4) obtained in the tests of the pig *E*. *bieneusi* populations implied an excess of low frequency polymorphisms, as would be expected from a recent population expansion.

We also calculated *I*^S^_A_ and compared the values of *V*_D_ (variance of pairwise differences) and *L* (the 95% critical value for *V*_D_ relative to the null hypothesis of panmixia) to assess the population structure of *E*. *bieneusi* using allelic profile data [34]. As presented in Table 5, significant positive *I*^S^_A_ values (at least 0.2733 in Qiqihar, *P*_MC_ < 0.001) were obtained for the overall and individual pig *E*. *bieneusi* populations. The value of *V*_D_ was greater than that of *L* for each data set as well. Thus, the populations tested were all in strong LD. Calculation of *I*^S^_A_ and comparison of *V*_D_ and *L* were also applied for the analysis of MLGs to avoid the possibility that LD would result from a clonal expansion of one or more MLGs which might mask the underlying equilibrium. The analysis showed the overall pig *E*. *bieneusi* population retained LD (*I*^S^_A_ = 0.1441, *P*_MC_ < 0.001; *V*_D_ > *L*) when the isolates with the same MLG were treated as one individual (Table 5). Pairwise intergenic analysis of the five loci using the allelic profile data revealed strong LD as well (S2 Table). We also estimated the effective migration rate (*Nm*) using the *F*_ST_ method. Pairwise analysis between geographic populations yielded *F*_ST_ values ranging from 0.327 to 0.506 and *Nm* values ranging from 0.24 to 0.48 (Table 6). Thus, geographic segregation of the isolates and limited gene flow occurred among the pig *E*. *bieneusi* populations from four cities.

**Table 5:** Results of linkage disequilibrium analysis based on allelic profile data from five genetic loci.

City/subpopulation	No.	Hd	*I*^S^_A_	*P*_MC_	*V*_D_	*L*	*V*_D_ > *L*
Changchun	34	0.6913 ± 0.0326	0.3763	< 0.001	2.62	1.1664	Y
Daqing	22	0.5740 ± 0.0267	0.6389	< 0.001	4.2961	1.4005	Y
Harbin	29	0.2739 ± 0.0737	0.4417	< 0.001	2.4508	1.1817	Y
Qiqihar	16	0.4717 ± 0.1335	0.2733	< 0.001	1.8621	1.1226	Y
Total (4 cities)	101	0.7986 ± 0.0406	0.3768	< 0.001	1.9338	0.8009	Y
Total (4 cities)^a^	44	0.8408 ± 0.0334	0.1441	< 0.001	1.0197	0.7022	Y
SP1	66	0.8366 ± 0.0351	0.1191	< 0.001	0.9725	0.7001	Y
SP2	39	0.1976 ± 0.0577	0.3729	< 0.001	1.8093	0.9471	Y
SP3	35	0.6971 ± 0.0605	0.4709	< 0.001	2.8327	1.0886	Y
SP4	41	0.5276 ± 0.0748	0.4866	< 0.001	3.3424	1.3644	Y
SP5	26	0.5292 ± 0.1023	0.2535	< 0.001	3.3424	1.3644	Y
Total (5 subpopulations)	207	0.8988 ± 0.0180	0.4666	< 0.001	1.2845	0.4563	Y
Total (5 subpopulations)^a^	105	0.9178 ± 0.0193	0.1577	< 0.001	0.6030	0.3821	Y

Hd: mean genetic diversity; *I*^S^_A_: standardized index of association calculated using the program LIAN 3.5; *P*_MC_: significance for obtaining this value in 1000 simulations using the Monte Carlo method; *V*_D_: variance of pairwise differences; *L*: 95% critical value for *V*_D_; *V*_D_ > *L* indicates linkage disequilibrium.

^a^ Considering isolates with the same MLG as one individual.

**Table 6:** Pairwise genetic distance (*F*_ST_, lower diagonal, *P* < 0.001) and gene flow (*Nm*, upper diagonal) between *Enterocytozoon bieneusi* populations.

	Changchun	Daqing	Harbin	Qiqihar		SP1	SP2	SP3	SP4	SP5
Changchun		0.48	0.32	0.51	SP1		0.39	0.46	0.24	1.10
Daqing	0.344		0.24	0.46	SP2	0.391		0.38	0.30	0.36
Harbin	0.436	0.506		0.41	SP3	0.352	0.398		0.48	0.39
Qiqihar	0.327	0.353	0.379		SP4	0.515	0.454	0.342		0.20
					SP5	0.185	0.412	0.393	0.559	

Population genetic analysis was also conducted when the MLST data from 106 *E*. *bieneusi* isolates from humans and baboons were included. Significant LD and limited recombination were obtained in the analysis of a total of 207 isolates (Tables 4 and 5). The test of selective neutrality revealed a nonneutral structure for the population (Table 4). The negative Fs and D statistics (highlighted in bold at the bottom of Table 4) obtained from this test signified potential epidemic expansion of some MLGs and genetic subdivision.

### Subpopulations and population genetic analysis

**Fig 1:**
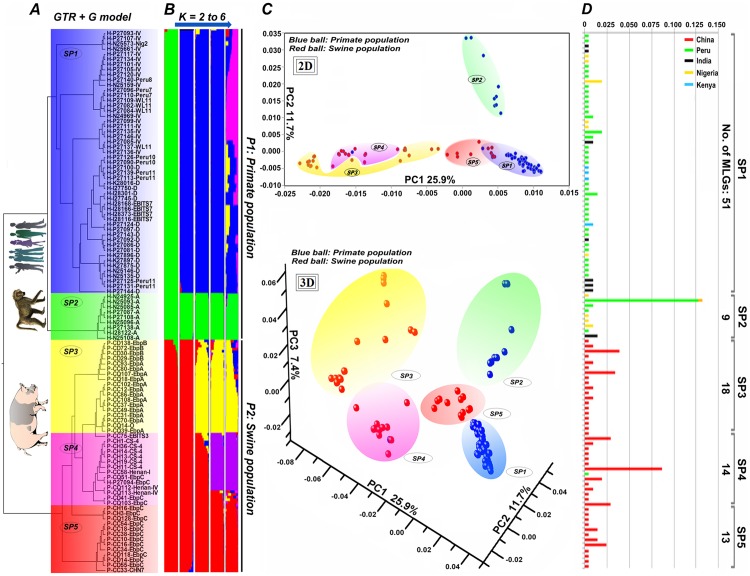
Comprehensive phylogenetic and structural analyses of *Enterocytozoon bieneusi* isolates from various hosts and locations. Panel A: Bayesian phylogenetic analysis of 105 unique multilocus genotypes (MLGs) from 207 isolates. Among them, 44 MLGs are from pigs, 61 from humans, and 4 from baboons, which are indicated by P, H, and B before the specimen numbers, respectively. The letters I, N, P, K, and C (C, D, H, and Q) followed indicate the isolates are from India, Nigeria, Peru, Kenya, and China (Changchun, Daqing, Harbin, and Qiqihar), respectively. ITS genotypes are labeled at the ends. Panel B: Subpopulation structure of all 207 *E*. *bieneusi* isolates. Various subpopulation patterns were obtained when different K values (2 to 6) were used. Panel C: Results of the principal coordinates analysis of 105 unique *E*. *bieneusi* MLGs based on pairwise distances. Red and blue balls represented the isolates from pig and primate populations, respectively. Panel D: Frequency (%) of *E*. *bieneusi* MLGs in the five subpopulations determined in structural analysis.

A Bayesian method was used to infer phylogenetic relationships among *E*. *bieneusi* isolates from pigs, humans, and baboons (Fig 1A). The isolates were divided into two major phylogenetic clusters (one includes 44 MLGs from pigs and 1 MLG from a Peruvian adult with HIV infection and the other one includes 56 MLGs from humans and 4 MLGs from baboons) (Fig 1A). Additional subdivision within the two clusters led to the formation of five genetically isolated subgroups (Fig 1A). The same clustering patterns appeared in the 3D image of PCoA with two main clusters (blue balls represent the isolates from humans and baboons and red balls from pigs) and five genetic subdivisions generated (Fig 1C). Considering the high concordance of the grouping patterns of MLGs formed in Bayesian inference and PCoA, we defined two known primate *E*. *bieneusi* subpopulations as SP1 and SP2 and three novel pig *E*. *bieneusi* subpopulations as SP3 to SP5. Subpopulations SP1 to SP5 contained 51, 9, 18, 14, and 13 MLGs derived from 66, 39, 35, 41, and 26 *E*. *bieneusi* isolates, respectively (Tables 2 and 4, Fig 1D). In general, subpopulations SP2 to SP4 had higher frequency of MLGs than SP1 and SP5 (Fig 1D).

We also performed substructure analysis based on allelic profile data using STRUCTURE. The initial run with K = 2 identified two major subclusters (Fig 1B). One subcluster in red included all isolates from pigs and one isolate from a human and the other subcluster in green contained the isolates from humans and baboons (Fig 1B), corresponding to the two major substructures generated in Bayesian inference and PCoA. The following runs at K = 3 and 4 yielded various intermediate patterns of population subdivision. The run at K = 5 showed the presence of five clear and robust subpopulations (Fig 1B). The isolates in each of the five subpopulations agreed with those in SP1 to SP5 except that several isolates from SP4 were clustered into SP5 (Fig 1). We also measured the alpha (α) values generated in the substructure analysis. When there are firm subdivisions in a population, the α values are held constant and commonly range from 0 to 0.2 in different runs. The runs at K = 2 to 5 in this study yielded consistent α values around 0.03, which supported the robustness of the substructure formation. However, when the analysis was performed at K = 6 or more, a fairly mixed and confused scene of clustering was observed (Fig 1B). Taken together, these results suggest that the run with K = 5 provided the best fit to our data.

Based on the results from Bayesian phylogeny, PCoA, and substructure analysis, it was apparent that five genetically isolated subdivisions were present in the total population. Distribution preference of the pig *E*. *bieneusi* isolates from Changchun and Daqing in SP3 and SP5 and those from Harbin and Qiqihar in SP4 suggested the presence of genetic segregation at some geographic level (S3 Table). Genetic diversity, intragenic LD, and Rms were measured based on multilocus sequences for each of the five subpopulations. Pairwise LD comparisons among the subpopulations showed that the clonality of *E*. *bieneusi* isolates in SP2 to SP4 was stronger than that in SP1 and SP5 (Table 4). In agreement with this, higher *I*^S^_A_ values were generated in the analysis of SP2 to SP4 than in SP1 and SP5 (Table 5). The measurement of population divergence among SP1 to SP5 was performed by the analysis of *F*_ST_ and gene flow (Table 6). SP1 and SP5 were shown to have a close genetic relationship (*F*_ST_ = 0.185 and *Nm* = 1.10) (Table 6). Nevertheless, in comparative analysis of SP1 or SP5 to any other three subpopulations, the *F*_ST_ values of at least 0.342 and *Nm* values of at most 0.48 indicated the presence of significant population differentiation and very limited gene flow.

**Fig 2:**
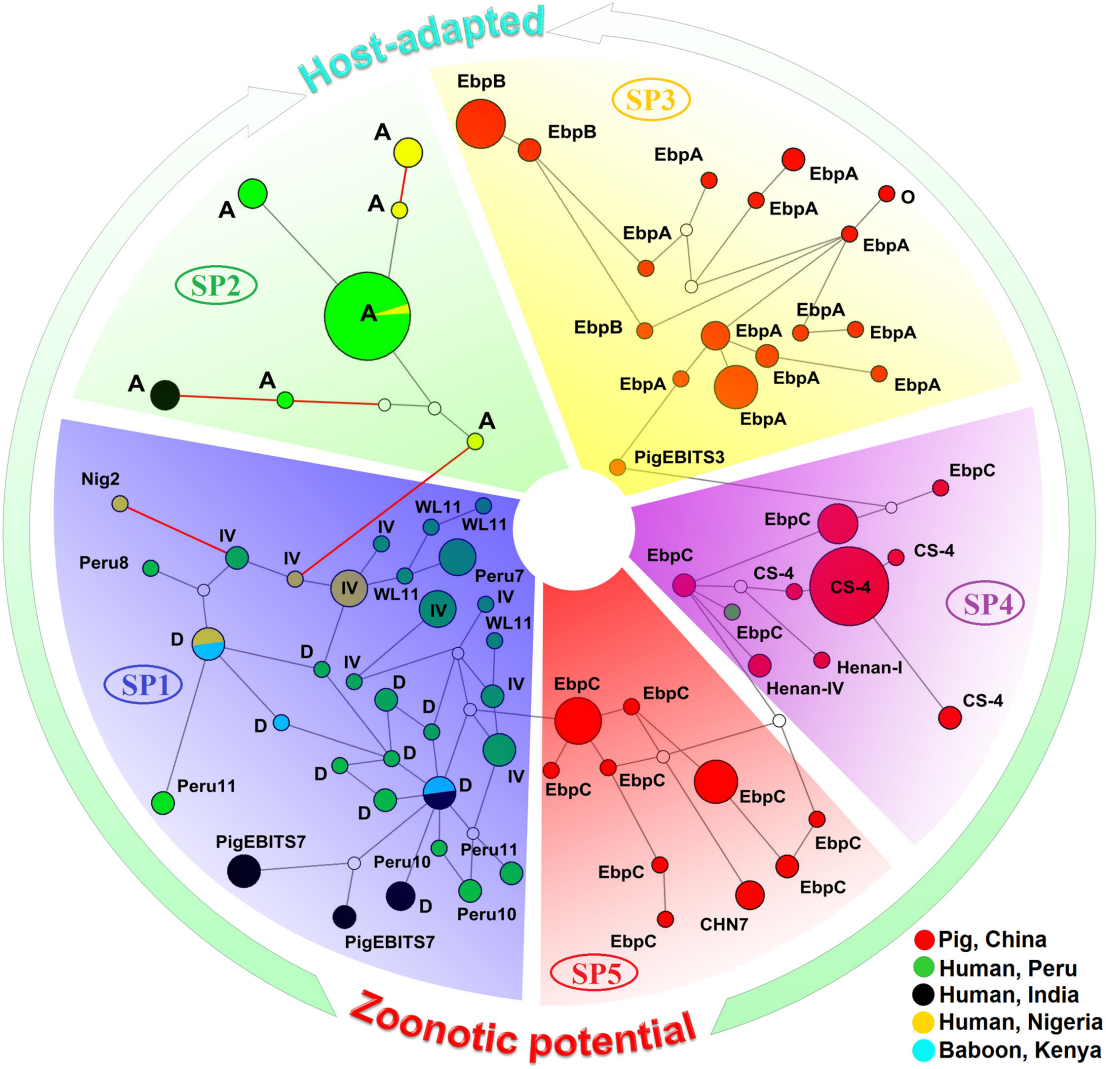
Median-joining network for inferring intraspecific phylogenies of 207 *Enterocytozoon bieneusi* isolates from pigs in China, humans in India, Nigeria, and Peru, and baboons in Kenya. The size of the circles is proportional to the frequency of each of the 76 multilocus genotypes obtained based on segregating sites. The red, black, blue, yellow, and green colors in circles represent the isolates from China, India, Kenya, Nigeria, and Peru, respectively. ITS genotypes are labeled besides the circles. The black branches connecting multilocus genotypes have a length proportional to the number of single-nucleotide polymorphisms (SNPs), while the red branches having pairwise differences greater than 12 SNPs are shortened for better presentation.

Median-joining networks were used to infer the relationships between MLGs (Fig 2). The analysis showed the existence of five clusters marked in blue, green, yellow, purple, and red, which corresponded to SP1 to SP5, respectively (Fig 2). As central haplotypes are generally considered possible ancestors of the peripheral ones [26,41], the MLGs in SP2 to SP4 might have derived from the central ones in SP1 and SP5 (Fig 2). In addition, high dimensional networks in SP1 suggested the presence of significant recombination (Fig 2).

## Discussion

Despite advances in defining *E*. *bieneusi* ITS genotypes from different hosts and geographical regions, the relationship between genotypes and phenotypic traits such as host specificity and zoonotic potential remains unclear [2,11]. Analysis of population structure can help us understand the epidemiology and evolution of parasites [42]. MLST analysis has provided substantial new insights into the population genetics of *E*. *bieneusi* in humans and non-human primates [26–28]. Herein, we evaluated intragenic and intergenic LD, *I*^S^_A_, neutrality, and Rms to determine the genetic structure and substructures in *E*. *bieneusi* populations from pigs using both sequence and allelic profile data from five genetic loci. The presence of strong LD and very limited recombination supported the existence of significant clonal structure in the overall and individual pig *E*. *bieneusi* populations from four cities, northeast China. In addition, as illustrated in Fig 2, the results of several neutrality tests suggest that selection acting in pig *E*. *bieneusi* populations has led to the expansion of several dominant MLGs, which might play a role in enhancing their adaptation to specific hosts. Two recent studies described that two other microsporidian species known to infect honeybees (*Nosema apis* and *Nosema ceranae*) were also under selective pressure and experienced a population expansion [43,44]. The estimates of *F*_ST_ and *Nm* and the distribution of the pig *E*. *bieneusi* isolates revealed the existence of geographic segregation among the cities surveyed.

Pigs are considered a potential reservoir for human microsporidiosis based on genotypic features of *E*. *bieneusi* isolates at the ITS locus [2,17,23]. The ITS genotypes of pig *E*. *bieneusi* isolates used for MLST analysis in this study all belong to phylogenetic Group 1 with zoonotic potential [2,17,23]. The ITS genotypes of primate *E*. *bieneusi* isolates used for comparative analysis are also Group 1 members [26,27]. These Group 1 isolates formed several genetically isolated subpopulations (two existing primate SP1 and SP2 and three novel pig SP3 to SP5) in MLST analysis of five genetic loci. SP1 to SP5 probably have different phenotypic traits as reflected in the distribution of *E*. *bieneusi* isolates in these subpopulations. As observed in Fig 1A and S4 Table, SP1 and SP5 are comprised mainly of the isolates pertaining to zoonotic ITS genotypes D, IV, and EbpC that are found in a wide range of hosts and regions around the world. In contrast, SP2 to SP4 contain mainly isolates belonging to ITS genotypes A, EbpA, and EbpB that have narrow host and geographic ranges.

The stronger LD and higher occurrence of specific MLGs observed in SP2 to SP4 than SP1 and SP5 suggest the presence of higher clonality of *E*. *bieneusi* isolates in the former three subpopulations. The high diversity of *E*. *bieneusi* isolates in SP1 and SP5 may enable responses to environmental challenges and adaptations to new hosts [45]. Thus, MLGs in SP1 and SP5 might be responsible for cross-species *E*. *bieneusi* infections and have zoonotic potential. This is supported by the broad host range of *E*. *bieneusi* ITS genotypes D, IV, and EbpC in the two subpopulations.

Reduced gene flow between primate SP1 and SP2 and between pig SP5 and SP3 might promote the emergence of advantageous haplotypes in SP2 and SP3 and allow these haplotypes to remain intact despite the possibility of recombination [46]. These processes might enable adaptation to specific host niches and initiate allopatric speciation [46]. In particular, this may have led to the adaptation of ITS genotype A in SP2 to humans and genotypes EbpA and EbpB in SP3 to pigs. Thus, MLGs in SP2 and SP3 are probably involved in host-specific colonization. SP2 was previously reported to have an epidemic population structure [26,27]. Likewise, genetic structure of SP3 with host-adapted features can also be considered to be epidemic. SP4 consists of *E*. *bieneusi* isolates belonging to the ITS genotypes CS-4, Henan-I, Henan-IV, PigEBITS3, and EbpC. The former four genotypes were shown to infect a very limited number of host species as shown in S4 Table, while EbpC has a wide host range. Thus, the subpopulation probably represents an evolutionary intermediate between SP3 with host-adapted traits and SP5 with cross-species capability. The placement of one human *E*. *bieneusi* isolate with ITS genotype EbpC in SP4 supported the zoonotic nature of some *E*. *bieneusi* isolates.

Population genetic analysis is an indirect but powerful way to assess reproductive modes that are often difficult to uncover in some microorganisms [42]. Sexual and asexual reproduction alternates in many protozoan species under environmental pressure [47]. It is generally accepted that microsporidia undergo sexual reproduction, whereas some species or genotypes possibly have switched to obligate asexuality [48]. This is supported by the existence of clonal population structure in SP1 and SP5 and epidemic population structure or host-adapted traits in SP3 to SP4. Although a sexual phase might be rare or virtually absent in some microsporidian species, there are footprints of recombination in their genomes [47,48]. This could be responsible for the small number of recombination inferred in subpopulations SP1 through SP5. Although limited recombination would not constitute sufficient evidence for the presence of sexuality in SP1 and SP5, the weakened LD and high levels of MLG diversity might facilitate *E*. *bieneusi* to cope with host variations and environmental challenges. The haplotype network suggests that clusters SP3/SP4 may have been derived from SP5 and that SP2 may have arisen from SP1. Thus, SP2 to SP4 with host-specific features might serve as recurrent transitions to asexuality from otherwise SP1 and SP5 with sexual potential and some of the isolates in SP2 to SP4 might have outcompeted or partially displaced their relatives in SP1 and SP5 under certain ecological conditions. This process would limit host range and result in the emergence of highly successful *E*. *bieneusi* genotypes. It has been suggested that some asexual microsporidian populations might originate independently several times from their sexual ancestors [48,49]. These results indicate that *E*. *bieneusi* might have a sexual phase in its life cycle, sex could be lost or cryptic, and the parasite could switch to obligate asexuality when the population structure becomes epidemic.

In conclusion, we have shown an overall clonal population structure of *E*. *bieneusi* in pigs. Combined with the MLST data from primate *E*. *bieneusi* isolates, five distinct subpopulations have been defined. Among them, the very strong LD and low genetic recombination are indicative of the epidemic or host-adapted characteristics of SP2 to SP4, whereas the weakened LD and higher genetic diversity in SP1 and SP5 may represent higher potential for cross-species transmission of *E*. *bieneusi* infections. These data demonstrate the existence of genetic structure within *E*. *bieneusi* ITS Group 1 and the evolutionary potential for adaptation to host species in some of the subpopulations. Nevertheless, additional MLST data from other hosts including humans in China are needed for in-depth assessment of the potential for zoonotic transmission, host adaptation, and population differentiation of *E*. *bieneusi* isolates in different hosts.

## Supporting Information

S1 Table Recombination detection. Recombination events assessed using the methods GENECONV, MaxChi, and SiScan.
(DOC)

S2 Table Intergenic linkage disequilibrium. Pairwise *P* values for intergenic linkage disequilibrium among genetic loci based on allelic profile data from pig *Enterocytozoon bieneusi* populations.
(DOC)

S3 Table Geographical distribution of specimens in genetic subdivisions. Proportion of the specimens from each city that belong to each of the three pig-derived *Enterocytozoon bieneusi* subpopulations.
(DOC)

S4 Table Distribution of ITS genotypes by host and location. Host range and geographical distribution of ITS genotypes of *Enterocytozoon bieneusi* referred in this study.
(DOC)
