# Supplementary material for: Clonal Evolution of Enterocytozoon bieneusi Populations in Swine and Genetic Differentiation in Subpopulations between Isolates from Swine and Humans
Source: PLoS Negl Trop Dis. 2016 Aug 26;10(8):e0004966. doi: 10.1371/journal.pntd.0004966 (PMC5001694; doi:10.1371/journal.pntd.0004966)
Supplement: S1 Table — Recombination events assessed using the methods GENECONV, MaxChi, and SiScan. (DOC) [file pntd.0004966.s001.doc]

**S1 Table. Recombination detection. Recombination events assessed using the methods GENECONV, MaxChi, and SiScan.**

| Location | Recombination events | | |
| --- | --- | --- | --- |
|  | GENECOV | MaxChi | SiScan |
| Changchun | 0 | 3 | 1 |
| Daqing | 0 | 10 | 0 |
| Harbin | 0 | 0 | 0 |
| Qiqihar | 1 | 2 | 1 |
| Total | 0 | 28 | 2 |
