# Supplementary material for: Clonal Evolution of Enterocytozoon bieneusi Populations in Swine and Genetic Differentiation in Subpopulations between Isolates from Swine and Humans
Source: PLoS Negl Trop Dis. 2016 Aug 26;10(8):e0004966. doi: 10.1371/journal.pntd.0004966 (PMC5001694; doi:10.1371/journal.pntd.0004966)
Supplement: S2 Table — Pairwise P values for intergenic linkage disequilibrium among genetic loci based on allelic profile data from pig Enterocytozoon bieneusi populations. (DOC) [file pntd.0004966.s002.doc]

**S2 Table. Intergenic linkage disequilibrium. Pairwise *P* values for intergenic linkage disequilibrium among genetic loci based on allelic profile data from pig *Enterocytozoon bieneusi* populations.**

|  | MS1 | MS3 | MS4 | MS7 | ITS |
| --- | --- | --- | --- | --- | --- |
| MS1 |  | + | + | + | + |
| MS3 | 0.000 |  | + | + | + |
| MS4 | 0.000 | 0.000 |  | + | + |
| MS7 | 0.000 | 0.000 | 0.000 |  | + |
| ITS | 0.000 | 0.000 | 0.000 | 0.000 |  |

+: significant difference in Markov chain analysis (*P* < 0.05).
