# Supplementary material for: Clonal Evolution of Enterocytozoon bieneusi Populations in Swine and Genetic Differentiation in Subpopulations between Isolates from Swine and Humans
Source: PLoS Negl Trop Dis. 2016 Aug 26;10(8):e0004966. doi: 10.1371/journal.pntd.0004966 (PMC5001694; doi:10.1371/journal.pntd.0004966)
Supplement: S3 Table — Proportion of the specimens from each city that belong to each of the three pig-derived Enterocytozoon bieneusi subpopulations. (DOC) [file pntd.0004966.s003.doc]

**S3 Table. Geographical distribution of specimens in genetic subdivisions. Proportion of the specimens from each city that belong to each of the three pig-derived *Enterocytozoon bieneusi* subpopulations.**

| Subpopulation (no. of specimens) | No. of specimens from each city (%) | | | |
| --- | --- | --- | --- | --- |
|  | Changchun | Daqing | Harbin | Qiqihar |
| SP3 (35) | 18 (51.4) | 12 (34.3) | 0 (0.0) | 5 (14.3) |
| SP4 (40) | 2 (5.0) | 1 (2.5) | 27 (67.5) | 10 (25.0) |
| SP5 (26) | 14 (53.8) | 9 (34.6) | 2 (7.7) | 1 (3.8) |
