# Supplementary material for: Clonal Evolution of Enterocytozoon bieneusi Populations in Swine and Genetic Differentiation in Subpopulations between Isolates from Swine and Humans
Source: PLoS Negl Trop Dis. 2016 Aug 26;10(8):e0004966. doi: 10.1371/journal.pntd.0004966 (PMC5001694; doi:10.1371/journal.pntd.0004966)
Supplement: S4 Table — Host range and geographical distribution of ITS genotypes of Enterocytozoon bieneusi referred in this study. (DOC) [file pntd.0004966.s004.doc]

**S4 Table. Distribution of ITS genotypes by host and location. Host range and geographical distribution of ITS genotypes of *Enterocytozoon bieneusi* referred in this study.**

| Genotype | Host (Location)^a^ | Reference |
| --- | --- | --- |
| A | Human (Cameroon, Gabon, Germany, Netherlands, Niger, Peru, Switzerland, Thailand), NHP (Kenya), Bird (CZE) |  |
| CHN7 | Pig (China) |  |
| CS-4 | Human (China), NHP(China), Cattle (China), Pig (China), Sheep (China) |  |
| D | Humans (Cameroon, China, Congo, England, Gabon, Malawi, Netherlands, Niger, Nigeria, Peru, Russia, Spain, Thailand, Vietnam), NHP (China, Kenya, Rwanda, USA), Cattle (Argentina, Korea, South Africa, USA), Horse (Colombia), Pig (China, CZE, Japan, USA), Cat (China), Dog (China, Portugal), Beaver (USA), Falcon (Abu Dhabi), Fox (China, Spain, USA), Mice (CZE, Germany), Muskrat (USA), Rabbit (Spain), Raccoon (USA), Raccoon dogs (China), Otter (USA), Wild boar (Austria, CZE, Slovak Republic), Bird (Iran), DSW (China), WW (China, Tunisia), WWTP (Spain) |  |
| EbpA | Human (China, CZE, Nigeria), NHP (China), Cattle (Germany), Horse (CZE), Pig (China, CZE, Germany, Japan, Poland, Switzerland, USA), Dog (China), Mice (CZE), Bird (CZE) |  |
| EbpB | Pig (China, Switzerland ) |  |
| EbpC | Human (China, Peru, Thailand, Vietnam), NHP (China), Cattle (Argentina), Pig (China, Germany, Japan, Switzerland, Thailand), Dog (China), Beaver (USA), Fox (USA), Muskrat (USA), Otter (USA), Raccoon (USA), Wild boar (Austria, CZE, Poland, Slovak Republic), DSW (China), Lake water (China), WW (China) |  |
| Henan-I | Human (China), Pig (China), Deer (USA), Wild boar (Austria), Lake water (China) |  |
| Henan-IV | Human (China), Pig (China), Chicken (China) |  |
| IV | Human (Cameroon, China, England, France, Gabon, Malawi, Netherlands, Niger, Nigeria, Peru, Uganda), NHP (China), Cattle (Korea, Portugal, USA), Cat (Colombia, Germany, Japan, Portugal), Dog (China, Colombia), Bear (USA), Ostrich (Spain), Snake (China), Squirrel (USA), Vole (USA), Lake water (China), WW (China, Ireland, Tunisia) |  |
| Nig2 | Human (Nigeria) |  |
| O | Human (Thailand), NHP (China), Pig (China, Germany, Thailand), Dog (China) |  |
| Peru7 | Human (Peru), NHP (Kenya) |  |
| Peru8 | Human (China, Malawi, Nigeria, Peru, Tunisia), NHP (China), Dog (China), Mice (CZE) |  |
| Peru10 | Human (Peru), Cat (Colombia ) |  |
| Peru11 | Human (China, Peru, Thailand), NHP (China, Kenya) |  |
| PigEBITS3 | Pig (China, Korea, Switzerland, USA) |  |
| PigEBITS7 | Human (China, Thailand), NHP (China), Pig (USA) |  |
| WL11 | Human (Peru), Cat (Colombia), Dog (Colombia), Fox (USA) |  |

^a^CZE: Czech Republic; NHP: nonhuman primates; DSW: drinking source water; WW: wastewater; WWTP: wastewater treatment plant.

**S4 References**

1. Li W, Kiulia NM, Mwenda JM, Nyachieo A, Taylor MB, et al. *Cyclospora papionis*, *Cryptosporidium hominis*, and human-pathogenic *Enterocytozoon bieneusi* in captive baboons in Kenya. J Clin Microbiol. 2011; 49: 4326-4329.

2. Santin M, Fayer R. Microsporidiosis: *Enterocytozoon bieneusi* in domesticated and wild animals. Res Vet Sci. 2011; 90: 363-371.

3. Kasickova D, Sak B, Kvac M, Ditrich O. Sources of potentially infectious human microsporidia: molecular characterisation of microsporidia isolates from exotic birds in the Czech Republic, prevalence study and importance of birds in epidemiology of the human microsporidial infections. Vet Parasitol. 2009; 165: 125-130.

4. Li W, Li Y, Li W, Yang J, Song M, et al. Genotypes of *Enterocytozoon bieneusi* in livestock in China: high prevalence and zoonotic potential. PLoS One. 2014; 9: e97623.

5. Jiang Y, Tao W, Wan Q, Li Q, Yang Y, et al. Zoonotic and potentially host-adapted *Enterocytozoon bieneusi* genotypes in sheep and cattle in northeast China and an increasing concern about the zoonotic importance of previously considered ruminant-adapted genotypes. Appl Environ Microbiol. 2015; 81: 3326-3335.

6. Yang Y, Lin Y, Li Q, Zhang S, Tao W, et al. Widespread presence of human-pathogenic *Enterocytozoon bieneusi* genotype D in farmed foxes (*Vulpes vulpes*) and raccoon dogs (*Nyctereutes procyonoides*) in China: first identification and zoonotic concern. Parasitol Res. 2015; 114: 4341-4348.

7. Karim MR, Dong H, Li T, Yu F, Li D, et al. Predomination and new genotypes of *Enterocytozoon bieneusi* in captive nonhuman primates in zoos in China: high genetic diversity and zoonotic significance. PLoS One. 2015; 10: e0117991.

8. Santin M, Fayer R. *Enterocytozoon bieneusi*, *Giardia*, and *Cryptosporidium* infecting white-tailed deer. J Eukaryot Microbiol. 2015; 62: 34-43.

9. Li W, Diao R, Yang J, Xiao L, Lu Y, et al. High diversity of human-pathogenic *Enterocytozoon bieneusi* genotypes in swine in northeast China. Parasitol Res. 2014; 113: 1147-1153.

10. Akinbo FO, Okaka CE, Omoregie R, Dearen T, Leon ET, et al. Molecular epidemiologic characterization of *Enterocytozoon bieneusi* in HIV-infected persons in Benin City, Nigeria. Am J Trop Med Hyg. 2012; 86: 441-445.

11. Wan Q, Lin Y, Mao Y, Yang Y, Li Q, et al. High prevalence and widespread distribution of zoonotic *Enterocytozoon bieneusi* genotypes in swine in northeast China: implications for public health. J Eukaryot Microbiol. 2016; 63: 162-170.

12. Sak B, Kvac M, Kvetonova D, Albrecht T, Pialek J. The first report on natural *Enterocytozoon bieneusi* and *Encephalitozoon* spp. infections in wild East-European House Mice (*Mus musculus musculus*) and West-European House Mice (*M. m. domesticus*) in a hybrid zone across the Czech Republic-Germany border. Vet Parasitol. 2011; 178: 246-250.

13. Karim MR, Wang R, Dong H, Zhang L, Li J, et al. Genetic polymorphism and zoonotic potential of *Enterocytozoon bieneusi* from nonhuman primates in China. Appl Environ Microbiol. 2014; 80: 1893-1898.
